# High morphological and genetic variabilities of *Ochlerotatus scapularis*, a potential vector of filarias and arboviruses

**DOI:** 10.1186/s13071-015-0740-6

**Published:** 2015-02-26

**Authors:** Vivian Petersen, Mariana Devicari, Lincoln Suesdek

**Affiliations:** Instituto Butantan, São Paulo, Brazil; Biologia da Relação Patógeno-Hospedeiro–Universidade de São Paulo, São Paulo, Brazil; Programa de Pós- graduação do Instituto de Medicina Tropical, Universidade de São Paulo, São Paulo, Brazil

**Keywords:** Plasticity, Population structure, Haplotype diversity, Morphological diversity

## Abstract

**Background:**

*Ochlerotatus scapularis* is a potential vector of filarias and arboviruses in the Neotropics. This species was once typically associated with sylvatic environments; however, cases of synanthropy and urbanization of this species have been increasingly reported in southeast Brazil. Despite the medical relevance of *Oc. scapularis*, its populational variability is not yet known. To our knowledge, this is the first report describing the morphological and genetic variabilities of this species.

**Methods:**

Population samples were characterized using the cytochrome oxidase subunit I (COI) mitochondrial gene and wing geometrics. Adult mosquitoes were collected from five sampling sites from remnants of the Atlantic forest embedded in the urban or rural areas of southeast Brazil.

**Results:**

In the 130 individuals analyzed, 46 COI haplotypes were detected. Haplotype diversity was high and ranged from 0.66 to 0.97. Six haplotypes were present in 61% of the individuals, whereas the remaining haplotypes were less frequent (39%). Wing shape was also highly polymorphic. Differentiation of populations across sampling sites according to genetic distances (F_st_ = −0.009 to 0.060) and morphological distances (Q_st_ = 0.47) indicated that populations were not identical. No correlations were noted for phenetic and genetic diversities (p = 0.19) or for genetic or phenetic distances with geographical distances (p = 0.2 and p = 0.18, respectively).

**Conclusions:**

Our study results suggest that *Oc. scapularis* has a rich genetic patrimony, even though its habitat is fragmented. Implications of such genetic richness with respect to vectorial competence, plasticity, and ability to exploit urbanized areas need to be further investigated.

**Electronic supplementary material:**

The online version of this article (doi:10.1186/s13071-015-0740-6) contains supplementary material, which is available to authorized users.

## Background

*Ochlerotatus scapularis* (Rondani 1848) is a Neotropical culicid widely distributed in the southeastern region of Brazil [1]. This mosquito species is frequently found in remnants of the Atlantic Forest biome embedded in large rural or urban locations [2].

Nonetheless, in southeast Brazil, this species has been reported to occur in sylvan rural and urban ecotones [3,4] that are inhabited by millions of people. Remarkably, *Oc. scapularis* can be synanthropic and enter human dwellings [5], and has been increasingly reported to inhabit urban parks. Although the larvae of *Oc. scapularis* preferentially develop in natural temporary pools of water in the soil, they might also exploit permanent natural or artificial water containers [6].

The concurrence of insects and humans in this case is particularly problematic, because this mosquito has vectorial competence for several human and animal pathogens such as Rocio virus [7]. *Oc. scapularis* was possibly involved in the transmission of Rocio encephalitis virus in the State of São Paulo (southeast Brazil). This human encephalitis epidemic [8] occurred between 1974 and 1978 in 20 municipalities of São Paulo, in which more than 1,000 people were infected, hundreds of deaths occurred, and an estimated 200 sequelae cases were reported.

Outside southeast Brazil, this mosquito might also be a vector of the worms *Dirofilaria immitis* [9] and *Wuchereria bancrofti* [10], and the viruses Venezuelan Equine Encephalitis [11], Ilheus [12], and Melao [13]. The epidemiological importance of this mosquito vector is reinforced by its gonotrophic discordance: as females might need more than one blood repast during a single gonotrophic cycle, this increases their contact with possible hosts [14].

Heretofore, some key investigations such as biological variability of *Oc. scapularis* had not yet been performed. Even the remarkable population polymorphisms regarding chaetotaxy and genitalia, which were empirically reported by Arnell [15] and Forattini [1], have not been quantitatively investigated.

To better understand the biology of *Oc. scapularis*, we, for the first time, investigated the populational variability of this species. We then compared population samples collected from some southeast Brazil municipalities with epidemiologically relevant conditions by using largely used genetic (mitochondrial COI gene) and morphological (wing geometry) markers.

## Methods

### Collection of mosquitoes

**Figure 1:**
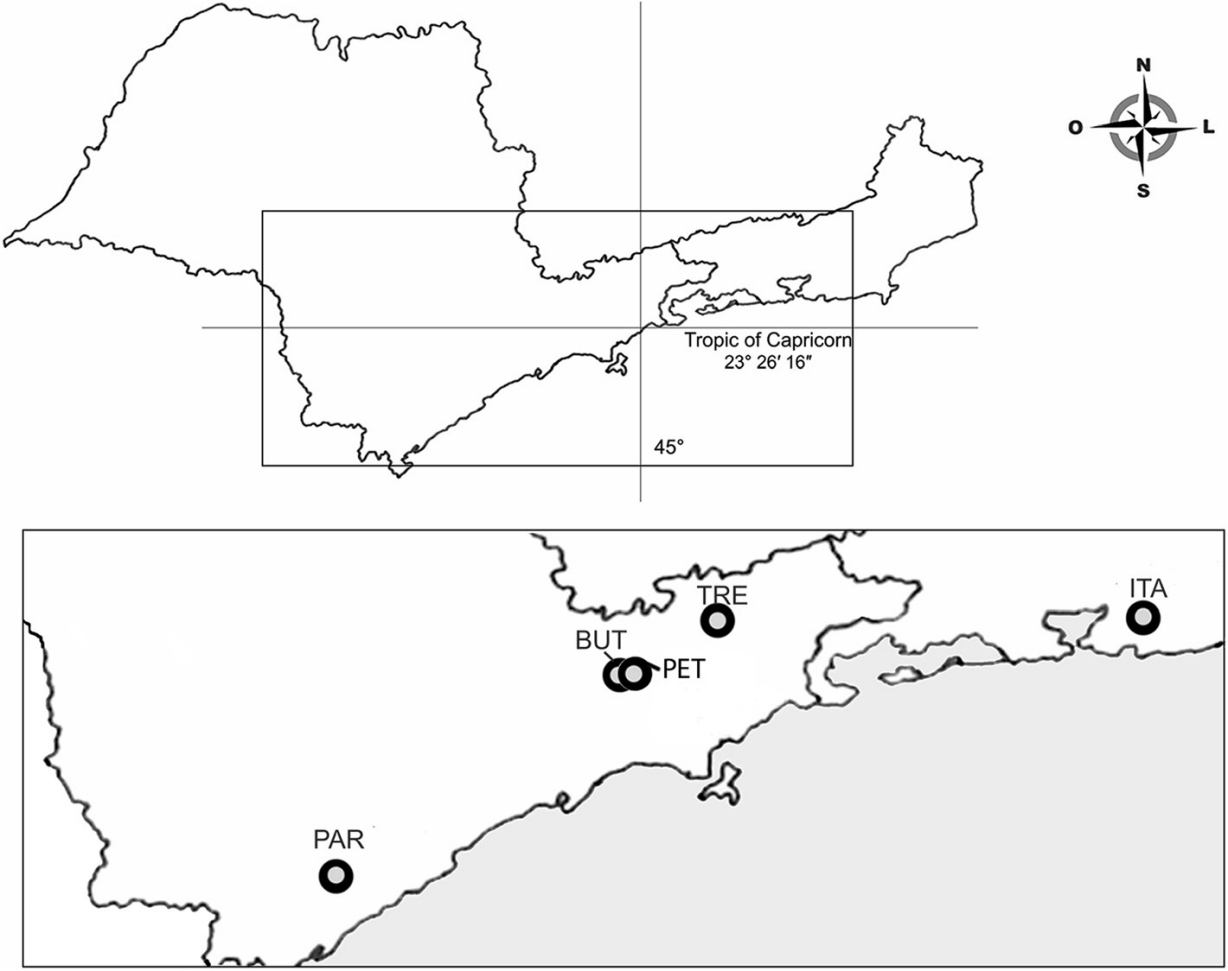
**Map of São Paulo and Rio de Janeiro states (above).** In detail, the four collecting sites of *Oc. scapularis* samples (below). More details shown in Table 1.

Sampling sites were located adjacent to the remnants of the southeastern Brazilian Atlantic Forest. Between 2007 and 2011, adult mosquitoes were collected using a portable insect aspirator from five locations (map with geographic coordinates shown in Figure 1): Parque Ecológico do Tietê (PET), Butantan neighborhood (BUT), Tremembé (TRE), Itaboraí (ITA), and Pariquera-Açu (PAR). The epidemiological relevance of the sampling locations is that some sites are urban parks located in areas inhabited by several millions of people, such as PET and BUT in São Paulo city (State of Sao Paulo). PAR was affected by the Rocio outbreak in 1974; it is a small urban locality surrounded by rural areas with buffalo ranches and rice paddies. TRE and ITA are located near rural areas inhabited by several thousands of people.

Owners of private properties and administrators of public parks authorized the mosquito collection. More collection details are shown in Table 1. Mosquitoes were identified down to the species level using taxonomic keys for female adult mosquitoes [1], and were individually placed in 1.5 ml microtubes with silica gel beads at room temperature until DNA and wing extraction.

**Table 1:** **Data of five samples** ***Oc. scapularis*** **collected in Brazil**

**Sample ID**	**Municipality**	**Locality**	**Number of samples (COI)**	**Number of samples (morphometrics)**	**Data**	**Geographic coordinates**
PET	São Paulo	Parque Ecológico do Tietê	34	29	April/2011	23° 29′15″S
						46° 31′90″W
BUT	São Paulo	Horto Instituto Butantan	10	39	February/2009	23°32′44″S
						46°43′39″W
ITA	Itaboraí	Pasture	36	30	March/2011	22°44′51″S
						42° 51′ 21″W
TRE	Tremembé	Fazenda Santa Cecília	29	30	November/2010	22°57′12″S
						45°32′28″W
PAR	Pariquera-Açu	Fazenda experimental	21	25	December/2007, March 2008,May 2009	24°42′ 37″S
						47°53′ 2″W

### DNA amplification and sequencing

A partial fragment of the mitochondrial gene cytochrome oxidase subunit I (COI) of the five populations was comparatively analyzed. Extraction of DNA was performed according to the Vidal & Suesdek [16]. The COI gene was chosen because it is a sensitive indicator of spatial genetic structure [17,18] and has been successfully used to study intraspecific genetic variation in insects [19-21]. Sample sizes in this analysis were as follows (Table 1): PET, ITA, TRE, PAR, and BUT. The COI gene was amplified using polymerase chain reaction (PCR) [22].

PCR amplicons of COI were electrophoresed in 1.0% agarose gel in TAE buffer (0.04 Tris buffer, 0.05 M sodium acetate, 0.01 M EDTA pH 8.0 (TAE). We used 2 μl of the PCR amplified, add 1 μl of 6× DNA Loading Dye (0.1% bromophenol and 30% glycerol) and 1 μl gel red. The primers 5′-LCO1490 GGTCAACAAATCATAAAGATATTGG-3′ and HC02198 5′-TAAACTTCAGGGTGACCAAAAAATCA-3′ were used for sequencing. For the final reaction 10 μl were added: 2 μl of 5× sequencing buffer, 3.6 pmol of primer (Forward or reverse), 0.25 of the Big Dye, 10–50 ng of DNA and ultrapure water. Reaction conditions were as follows: 96°C (15 s), 50°C (15 s) and 60°C (4 min) for 25 cycles [23]. For the sake of accuracy, amplicons of each individual were sequenced twice and high fidelity Platinum® *Taq* DNA Polymerase was used.

In order to quantify genetic polymorphism and compare across populations, the COI gene sequences were aligned in ChromasPro 2.4 [24]. Next, the number of haplotypes (H), haplotype diversity (h), and nucleotide diversity (π) were estimated using software DNAsp 5.0 [25]. The neutrality tests Tajima’s and Fu’s Fs were performed using Arlequin 3.11 [26] for testing whether the mutations were neutral. Fu’s Fs test is very sensitive to population expansion. The genetic differentiation index (F_st_) test for short divergence time was performed using Arlequin 3.11 software. The genetic distance among populations of *Oc. scapularis* was calculated using the Kimura 2-parameter (K2P) by using software Mega version 5.1 [27]. A haplotype network of COI sequences was constructed using a parsimony framework by using TCS 1.21 software [28], and the network was edited using the Image program. The cladogram of haplotypes was constructed using Mr Bayes software with 10,000,000 generations.

### Morphometric analyses of the wing

Wings are good biological markers owing to its bidimensionality and heritability [29]. Geometric morphometrics was chosen to describe the wings of *Oc. scapulars* because it is a sensitive and cheap technique, which has been increasingly used to describe inter and intraspecific variability [16,29-32].

The geometry of (right) wings of females from the five populations was comparatively analyzed. Samples sizes were PET, ITA, TRE, PAR, and BUT (Table 1). Geometric morphometrics reported by Vidal & Suesdek [18] were used. Images of the wings were captured using a Leica 320 digital camera coupled with a Leica S6 stereoscope. We digitized 18 landmarks using TpsDig V.1.40. Procrustes superimposition of raw positional coordinates and shape coordinates (to assess the wing shape) was calculated using computer programs TpsUtil 1.29 and TpsRelw 1.39 [33]. The principal components (PCs) of shape coordinates and discriminant analyses of the samples based on PCs were calculated using Morphoj 1.05 software [34] for evaluating the populational degree of similarity among the five populations studied.

Morphological diversity was estimated using the “amount of dispersion” of individuals in the morphospace of PCs. Such dispersion was calculated as follows: plots in the morphospace of PCs (each corresponding to a single mosquito) were digitized using TpsDig software (as performed for wings) in order to register their positional coordinates in an imaginary Cartesian plane. The centroid size of a set of individuals (a population) was calculated using TpsRelW software. Such centroid size was then considered as an indicator of the morphological diversity of a population. Theoretically, the amount of dispersion of individuals (of a single set) in the morphospace of PCs is proportional to the morphological variability of that set (see Additional file 1: Figure S1 for more details).

After the allometric effect on wing shape was estimated and removed, wings were compared among populational samples by using discriminant analysis [35]. Mahalanobis distances (p-value from non-parametrical tests with 10,000 permutation rounds) were used to construct phenetic trees by using the algorithm Neighbor-Joining (PAUP [36] and COV softwares [37]).The correlation between geographic and phenetic distances was also statistically tested using Pearson’s correlation in Statistica 7 [38].

Wing images of other species (*Aedes aegypti, Aedes albopictus, Anopheles cruzii, Anopheles homunculus, Culex nigripalpus, and Culex quinquefasciatus*) were also included in one of the comparative morphometric analyses. Those species were collected with a similar procedure of *Oc. scapularis* in locations mentioned in the Additional file 2: Table S1.

## Results

### Genetic analyses

We obtained a 448-bp fragment that is located in the barcode region of the COI gene and is also known to be highly variable and indicative of populational genetic polymorphisms [39,40]. In the 130 analyzed individuals, 46 COI haplotypes (GenBank accession number KM115416-61) were identified; 38 sites in the 448-bp fragment were variable. The statistical analysis of the genetic parameters is summarized in Table 2. The overall haplotype diversity was high (0.91), whereas the genetic pairwise distances among populations (Kimura 2-parameter) were low, ranging from 0.0054 to 0.0091 (Table 3).

**Table 2:** **Summary statistics for polymorphisms of five** ***Oc. scapularis*** **populations**

	**N**	**Unique haplotypes**	**H**	**h (SD)**	**π (SD)**	**DT**	**FS**
PET	34	5	16	0.8841	0.007182	−1.26855	−6.62988*
ITA	36	8	18	0.9238	0.008977	−0.92875	−6.92782*
TRE	29	12	23	0.9704	0.008856	−1.24190	−19.15227*
PAR	21	3	9	0.8476	0.006392	−0.52875	−0.62766
BUT	10	3	5	0.6667	0.004395	−0.85010	−0.97655

N= Number of sequences used; H= Number of haplotypes, h (SD) = Haplotype diversity; π (SD)= Nucleotide diversity, DT = Tajima’s D test; FS = Fu’s Fs test statistic; * significant values at p< 0.05.

**Table 3:** **K2P Genetic distance between five** ***Oc. scapularis*** **populations**

	**PET**	**ITA**	**TRE**	**PAR**
ITA	0.0086	-		
TRE	0.0081	0.0091	-	
PAR	0.0067	0.0079	0.0075	-
BUT	0.0069	0.0074	0.0069	0.0054

**Figure 2:**
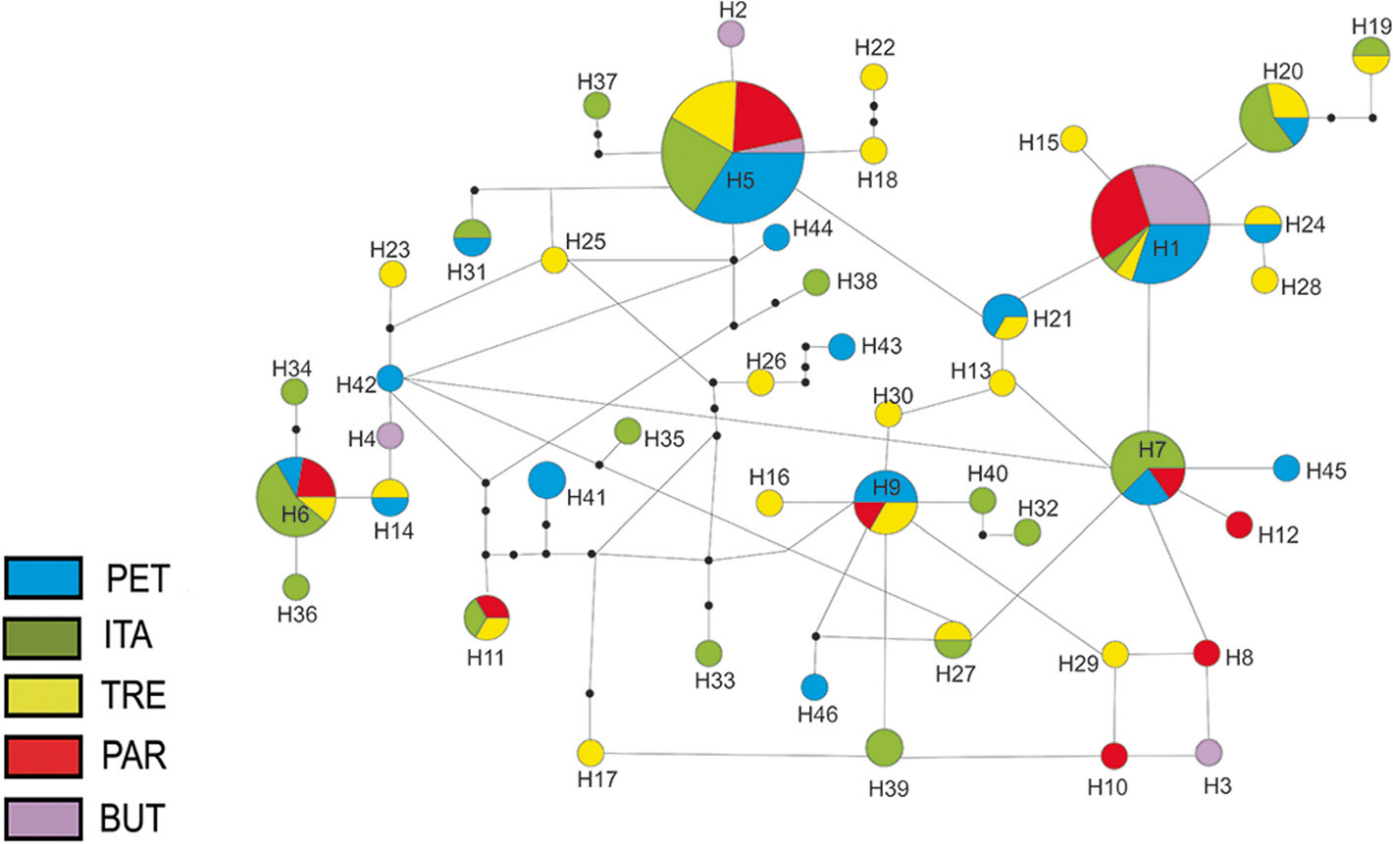
**Haplotype networks based on COI gene in five populations of** ***Oc. scapularis*** **from Brazil.** The size of each circle or circular sector is proportional to the number of individuals sharing that haplotype; the smallest circle corresponds to a single individual.

The similarities among haplotypes were depicted in a network (Figure 2). Two haplotypes (H1 and H5), which were the most frequent, were shared by all population samples and were present in 37% of the individuals, whereas the other moderately frequent haplotypes (H6, H7, H9 and H20) were shared by 3–4 populations and were present in 23% of the individuals. The remaining 40 haplotypes appeared in low frequencies and were mostly exclusive to one population (1–3 individuals each).

F_st_ scores ranged from −0.009 to 0.060 and were consistent with a weak or no population structure (Table 4), and the highest estimated divergence (between ITA and BUT) was only moderate. Some F_st_ scores, although negative, were not significantly different from zero.

**Table 4:** **Pairwise F**_**st**_ **estimates between five** ***Oc. scapularis*** **populations**

	**PET**	**ITA**	**TRE**	**PAR**
ITA	0.04433	-		
TRE	−0.00946	0.00328	-	
PAR	−0.02371	0.01580	−0.01997	-
BUT	−0.01310	0.06021*	0.01052	−0.02957

*Indicates moderate structure (P value significant at <0.05).

### Morphometric analyses

**Figure 3:**
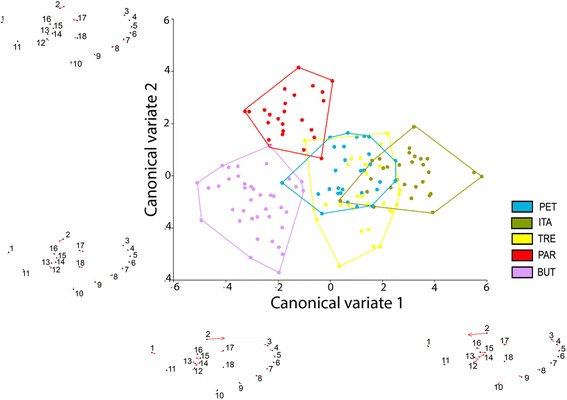
**Morphological space of canonical variates resulting from the comparison among the five populations of** ***Oc. scapularis*** **.** The 4 vectorial diagrams along the morphospace are “Thin plate splines” resulting from regression of canonical scores against shape components.

Discriminant analysis of the shape PCs, represented by canonical variables, revealed a complete discrimination between ITA and BUT populations and slight differences among the other populations: polygons (populations) in the morphospace overlapped partly (Figure 3). Although the slight populational differentiation noted, Mahalanobis distances were statistically significant (nonparametric p< 0.0001) for all pairwise comparisons, which is ordinarily indicative of a population structure. The Q_st_ estimator of phenetic populational differentiation scored 0.47, a value as high as to corroborate the presence of population structure [29].

**Figure 4:**
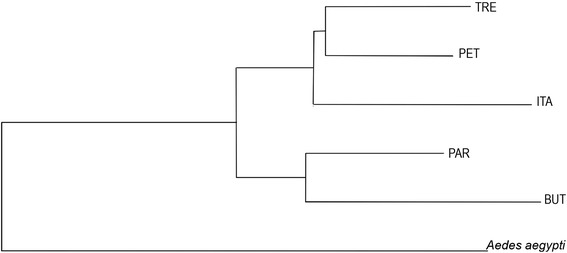
**Neighbor-Joining dendrogram of Mahalanobis pairwise distances between the five populations of** ***Oc. scapularis.***

The phenogram of Mahalanobis distances determined on the basis of wing shape (Figure 4) and results of correlation analysis did not reveal any significant correlation between phenetic and geographic distances (r = 0.46 p = 0.18).

**Figure 5:**
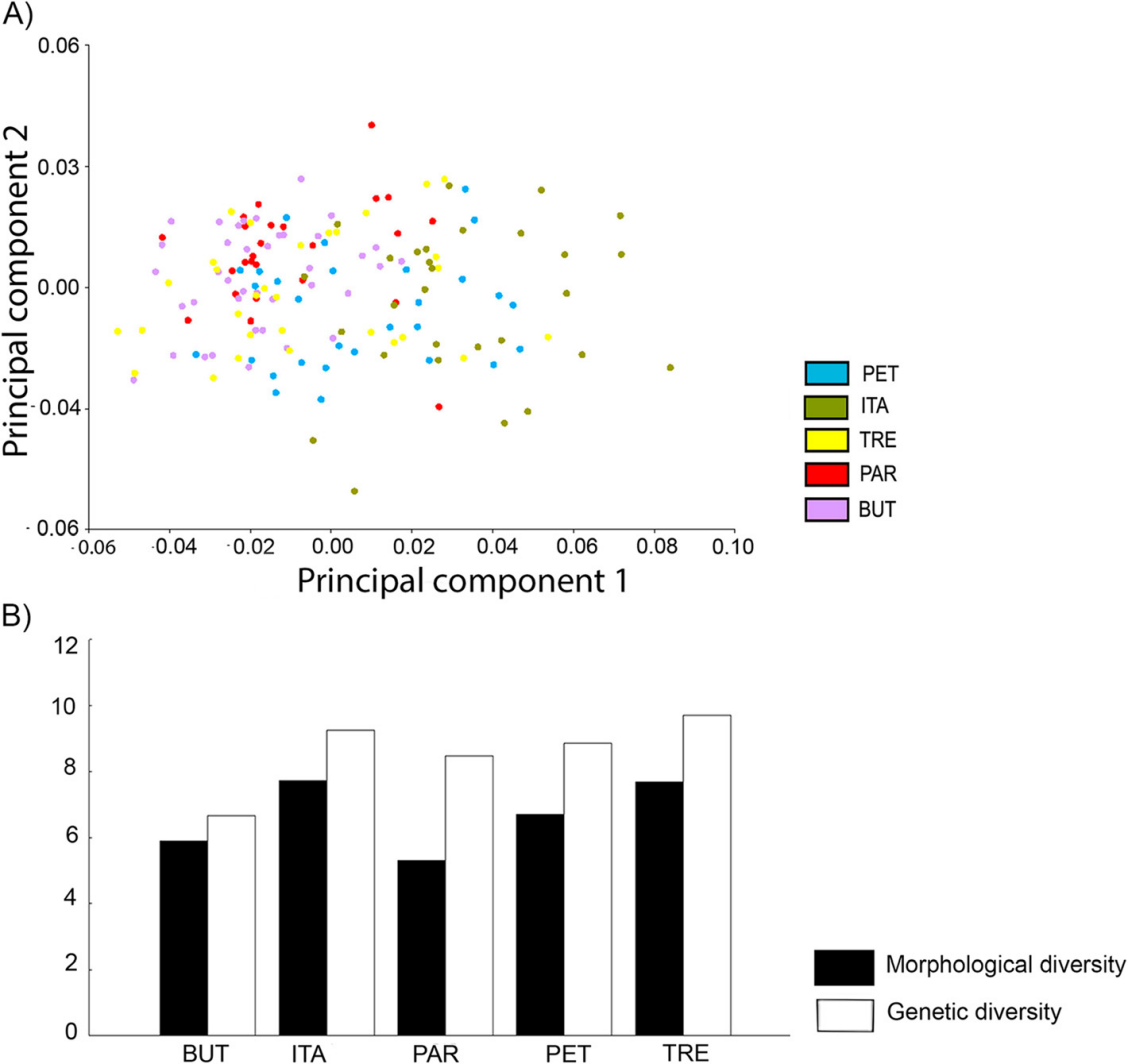
**A Morphological space of 1st and 2nd shape principal components (PCs) of five populations of** ***Oc. scapularis*** **. B** Comparison between haplotype diversity and morphological diversity of five populations.

**Figure 6:**
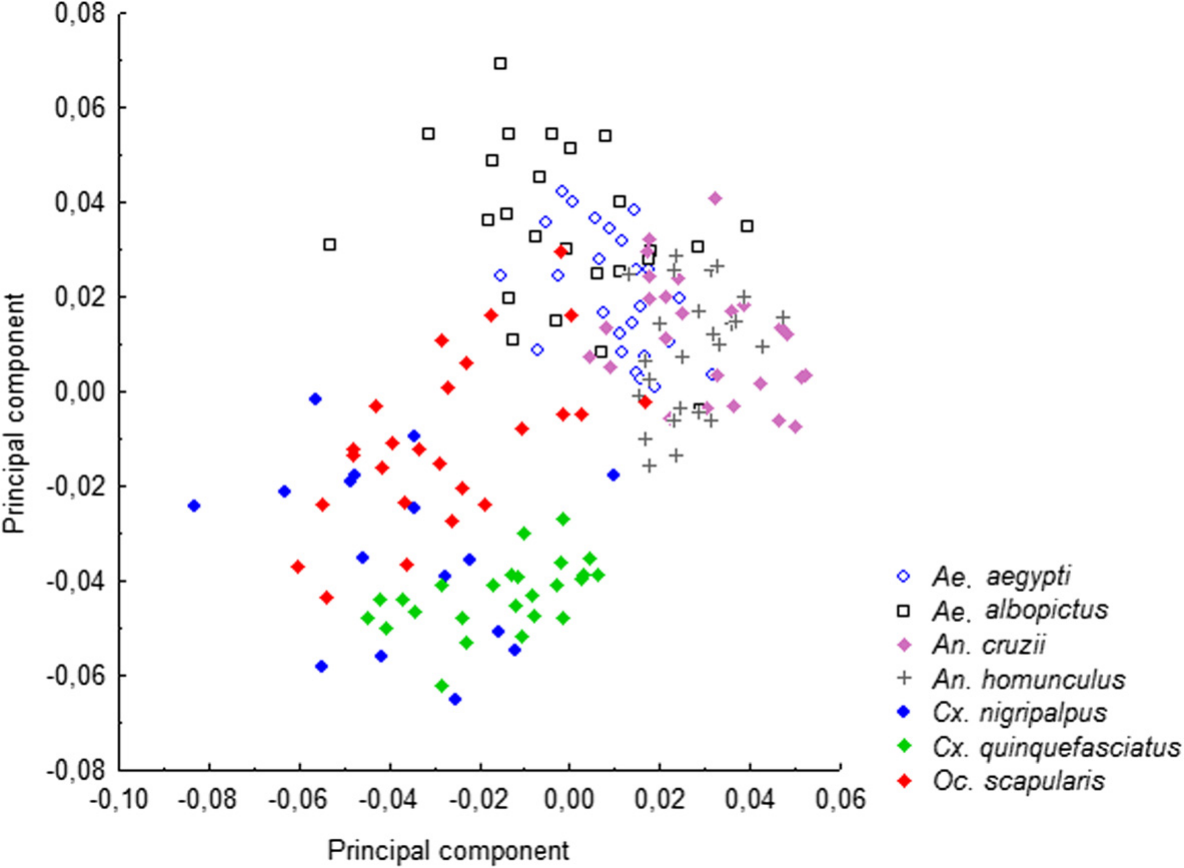
**Comparative Morphological diversity of** ***Oc. scapularis, Ae. aegypti, Ae. albopictus*** **,** ***An. cruzii*** **,** ***An. homunculus*** **,** ***Cx. nigripalpus and Cx. quinquefasciatus.***

Figure 5 shows that morphological diversity (estimated using the size of the spread of individuals in the morphospace of PCs) was variable, but its scores varied less than those of genetic diversity (estimated using haplotype diversity). No significant correlation was found between these 2 diversity estimators (r = 0.69, p = 0.19). Morphological diversity of *Oc. scapularis* was also high when compared to those of 6 other culicid species (Figure 6).

## Discussion

### Variability

A high haplotype diversity (mean = 0.91) was noted in the five populations. Such high polymorphism might indicate that these populations have not been recently affected by a bottleneck, despite the dramatic fragmentation of the Atlantic Forest during the past 300 years [41]. Similarly, the negative values of Tajima’s and Fu’s Fs for all populations suggest that these populations might be expanding or have a selective sweep of their COI gene [42,43]. Similar patterns and interpretations were reported in a population study of the culicid *Anopheles lesteri*, which was determined using COI gene polymorphism [44]. These authors obtained only negative values in neutrality tests and suggested that the populations under study were most likely undergoing expansion.

*Oc. scapularis* is naturally infected with *Wolbachia* (Noguera & Suesdek, unpublished data), and the presence of this endosymbiotic bacterium could decrease the number of haplotypes, as documented by Morais et al*.* [45] in *Culex quinquefasciatus.* However, the COI gene in *Oc. scapularis* still displays a high rate of polymorphism. Another Neotropical species with high genetic polymorphism is *An. cruzii* Dyar & Knab, which presented 60 haplotypes in 96 individuals, with a haplotypic diversity = 0.98 [46]. Nevertheless, large haplotype diversity is not commonly reported in Culicidae. For example, only 12 COI haplotypes were found in 88 individuals of *An. darlingi* Root (a predominant species in South America), from five locations in Colombia [47]. Another example is *Ae. albopictus* (Skuse), a species evolutionarily close to *Oc. scapularis*, which has only 16 haplotypes from 377 specimens [48]. Thus, *Oc. scapularis* appears to be a highly genetically diverse species. We find it improbable that such discrepancy is because of experimental errors or sampling biases because amplifications/sequencing were replicated and the high genetic diversity is consistent with the high morphological diversity observed.

In addition, the morphological diversity of *Oc. scapularis* was high in all populations. Even other Neotropical sylvatic species (*An. homunculus*, *An. cruzii*, *Cx. nigripalpus*, and *Ae. albopictus*) showed lesser diversity than *Oc. scapularis*.

The removal of both isometric and allometric effects of size permitted us to assess the wing geometry almost purely based on its shape. Considering that wing shape of culicids is mainly determined by quantitative polygenic heritage [29] and was highly variable in *Oc. scapularis*, we believe that wing shape may be a morphological indicator of genetic variability. A similar interpretation was given in studies of *Culex coronator* Dyar & Knab [30].

### Population structure

Bayesian phylogenetic inference derived using COI data (data not shown) failed to show reliable clusters of populations, and thus, do not suggest the presence of a population structure. Although the interpretations of the phylogenetic relationships among haplotypes cannot yet be inferred, the major haplotypes H1 and H5 seem to be closer to a common ancestor. All or most of the populations shared H1 and H5, and the interpopulation differentiation was low. In fact, all F_st_ scores were below 0.07, and gene flow was estimated to be significant. The absence of correlation between genetic and geographical (p = 0.2) distances is also consistent with a weak population structure.

As H1 and H5 were the only haplotypes shared by all populations, they might be considered as the remnants of an ancestral polymorphism. This might be an alternative explanation to interpopulation migration, as revealed in studies of the retention of mitochondrial haplotypes in *Anopheles arabiensis* Patton and *Anopheles gambiae* Giles [49].

Remarkably, suitable habitats for *Oc. scapularis* (rural areas, forest borders, and parks) are discontinuous across southeast Brazil. Even in the populational sample obtained from BUT, where collections were made from the smallest forest remnant (~40,000 m^2^) surrounded by densely urbanized neighborhood, high genetic and phenetic diversities were noted. The observed pattern of population similarity could be explained by the retention of ancestral polymorphisms or current passive migration, or might merely be a result of the colonization history. Among Culicidae, another species that has maintained interpopulation similarity despite wide geographical distribution and habitat discontinuity, is the malaria vector *An. darlingi* [50].

Wing shape characteristics provided some evidence of an incipient population structure, coherently supported by canonical variates morphospace, Mahalanobis distances, and Qst. The different Mahalanobis distance scores can be interpreted as different degrees of structuration, but the underlying factors to this heterogeneity are still unknown. The phenogram of morphological distances showed that there was no correlation between phenetic and geographic distances. For instance, ITA and PET clustered together despite being the farthest localities. As this analysis is not phylogenetic we could not study in detail the evolutionary relationships between groups, but we can assume that the evolution of wing shape did not follow an isolation by distance pattern.

### Markers combined

Results from the analyses of wing shape and COI variability were consistent but not identical. While both markers indicated slight populational differentiation, divergence derived from wing shape was more conspicuous. In fact, no statistical correlation was noted between the phenetic and genetic distances (r = 0.69, p = 0.19). Apparently, wing shape is less evolutionarily stable than the COI gene, as previously hypothesized by other authors [50]. Despite the suspected congruence between genetics and phenetics, the current results reinforce the idea that wing geometry bears informative biological markers that are sensitive to microevolutionary processes and can be considered as preliminary indicators of population structure. Other authors also share this point of view [16,50,51].

Accordingly, wings and COI revealed high morphogenetic variability (both intra and interpopulational). This was unexpected because the Atlantic Forest, the original habitat of this species, has been highly degraded in the past three centuries. This finding led us to pose a new hypothesis: Such biological richness might contribute to plasticity or a broad adaptation capacity of this species. This conjecture is in accordance with the findings from previous studies on *Oc. scapularis,* suggesting that they have vectorial competence [8,11], ability to breed in artificial containers [6], tendency to occur in urban locations (PET and BUT samples), and the potential to exhibit endophily and synanthropy when present in rural or semi-rural environments [48]. Further investigations are necessary to confirm our proposed hypothesis.

## Conclusion

We found many morphogenetic polymorphisms in the studied populations of *Oc. scapularis*, despite the species being from a fragmented habitat. Wing shape denoted incipient population structure and showed that this species is morphologically more diverse than other sylvatic Neotropical culicids. Unfortunately, studies on *Oc. scapularis* are still rare despite its possible epidemiological relevance. Future investigations need to be performed to better understand the structure and dynamics of the biological variability of this species as well as the possible implications of this variability on the plasticity, synanthropy, and vectorial capacity of this species.

## Additional files

Additional file 1: Figure S1. Morphological diversity estimated using the “amount of dispersion” of individuals in the morphospace of PCs. Each plots in the morphospace of PCs corresponding to a single mosquito were digitized using TpsDig software in order to register their positional coordinates in an imaginary Cartesian plane. The centroid size of a set of individuals (a population) was calculated using TpsRelW software. Such centroid size was then considered as an indicator of the morphological diversity of a population. Theoretically, the amount of dispersion of individuals (of a single set) in the morphospace of PCs is proportional to the morphological variability of that set. Subgraphs upper left of total PCs graph represents dispersion points of PAR population; Subgraphs upper right of total PCs graph represents dispersion point of ITA populations; Subgraphs lower left of total PCs graph represents dispersion points of BUT populations; Subgraphs lower of total PCs graph represents dispersion points of PET populations; Subgraphs lower right of total PCs graph represents dispersion points of PET populations; Subgraphs lower right of total PCs graph represents dispersion points of TRE populations. Additional file 2: Table S1. Data of samples of *Aedes aegypti, Aedes albopictus, Anopheles cruzii, Anopheles homunculus, Anopheles strodei, Anopheles nigripalpus*, and *Culex quinquefasciatus* collected in Brazil.

## Abbreviations

BUT: Butantan
COI: Cytochrome oxidase subunit I
DT: Tajima’s D test
FS: Fu’s Fs test
F_st_: The genetic differentiation index
H: Number of haplotypes
H: Haplotype diversity
ITA: Itaboraí
PAR: Pariquera-Açu
PET: Parque ecológico do Tietê
Q_st_: Quantitative trait differentiation index*.*
TRE: Tremembé
π: Nucleotide diversity
