# Supplementary figures and images for: High morphological and genetic variabilities of Ochlerotatus scapularis, a potential vector of filarias and arboviruses
Source: Parasit Vectors. 2015 Feb 26;8:128. doi: 10.1186/s13071-015-0740-6 (PMC4357162; doi:10.1186/s13071-015-0740-6)

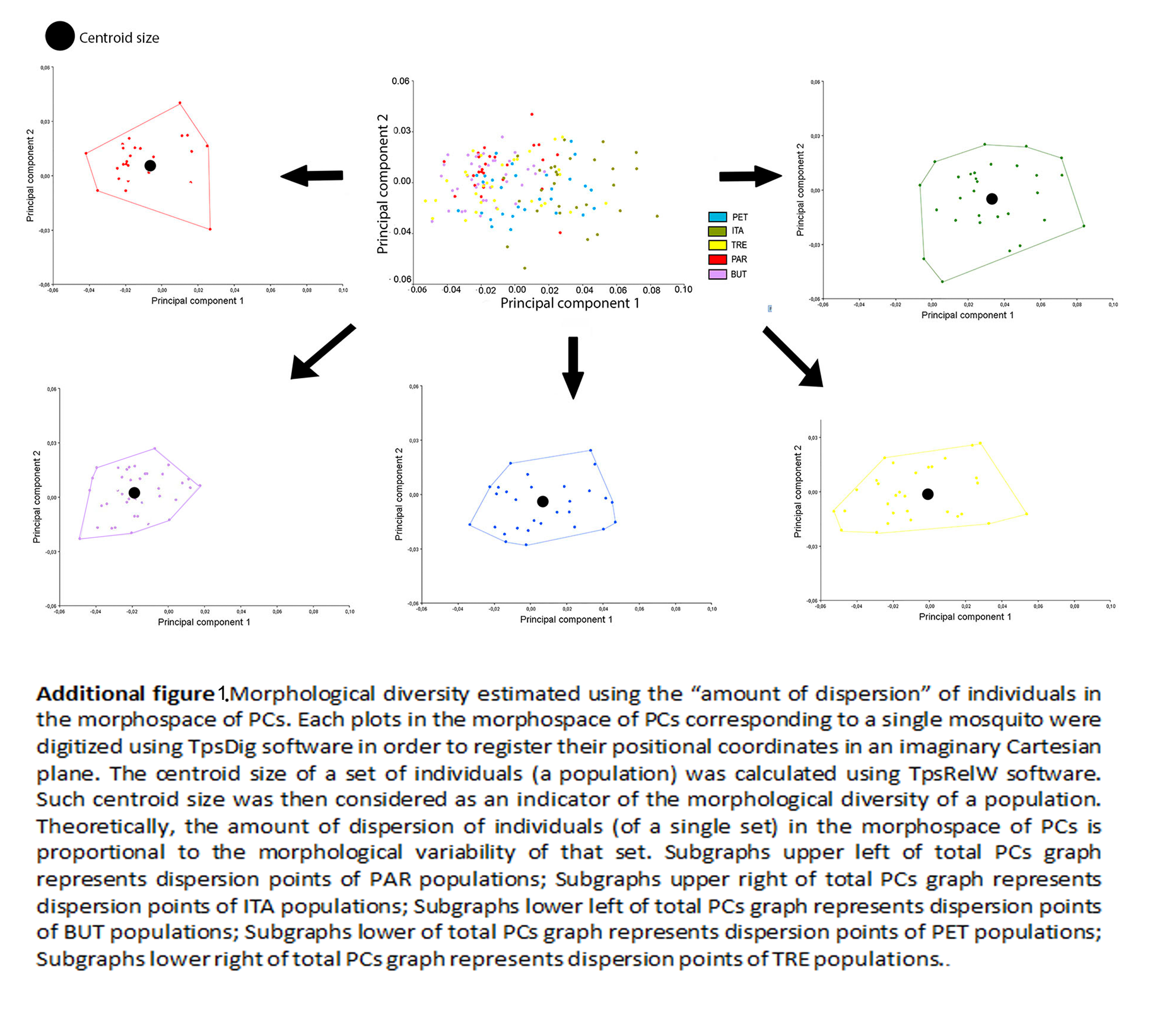

Supplement: Additional file 1: Figure S1. — Morphological diversity estimated using the “amount of dispersion” of individuals in the morphospace of PCs. Each plots in the morphospace of PCs corresponding to a single mosquito were digitized using TpsDig software in order to register their positional coordinates in an imaginary Cartesian plane. The centroid size of a set of individuals (a population) was calculated using TpsRelW software. Such centroid size was then considered as an indicator of the morphological diversity of a population. Theoretically, the amount of dispersion of individuals (of a single set) in the morphospace of PCs is proportional to the morphological variability of that set. Subgraphs upper left of total PCs graph represents dispersion points of PAR population; Subgraphs upper right of total PCs graph represents dispersion point of ITA populations; Subgraphs lower left of total PCs graph represents dispersion points of BUT populations; Subgraphs lower of total PCs graph represents dispersion points of PET populations; Subgraphs lower right of total PCs graph represents dispersion points of PET populations; Subgraphs lower right of total PCs graph represents dispersion points of TRE populations. [file 13071_2015_740_MOESM1_ESM.tiff]
