# Supplementary material for: High morphological and genetic variabilities of Ochlerotatus scapularis, a potential vector of filarias and arboviruses
Source: Parasit Vectors. 2015 Feb 26;8:128. doi: 10.1186/s13071-015-0740-6 (PMC4357162; doi:10.1186/s13071-015-0740-6)
Supplement: Additional file 2: Table S1. — Data of samples of Aedes aegypti, Aedes albopictus, Anopheles cruzii, Anopheles homunculus, Anopheles strodei, Anopheles nigripalpus, and Culex quinquefasciatus collected in Brazil. [file 13071_2015_740_MOESM2_ESM.docx]

**Additional file 2**. Data of samples of *Aedes aegypti, Aedes albopictus, Anopheles cruzii, Anopheles homunculus, Anopheles strodei, Anopheles nigripalpus*, and *Culex quinquefasciatus* collected in Brazil.

| Sample | Municipality | Number  of sample  Morphometric | Data | Geographic coordinates |
| --- | --- | --- | --- | --- |
| *Aedes aegypti* | São José do Rio Preto | 25 | April/2011 | 20º82’02”S  49º37’97”W |
| *Aedes albopictus* | Campinas | 25 | October /2011 | 22º90’64”S  47º06’16”W |
| *Anopheles cruzii* | Cananéia | 25 | July/2001 | 25º02’44”S  47º93’23”W |
| *Anopheles homunculus* | Cananéia | 25 | August/2011 | 25º02’44”S  47º93’23”W |
| *Anopheles strodei* | Frutal | 25 | February/ 2014 | 20º01’97”S  48º91’94”W |
| *Anopheles nigripalpus* | São Paulo | 16 | April/2007 | 23º29’15”S  46º31’90”W |
| *Culex quinquefasciatus* | São Paulo | 25 | September/2008 | 23º29’15”S  46º31’90”W |
